# ‘Maternal deaths should simply be 0’: politicization of maternal death reporting and review processes in Ethiopia

**DOI:** 10.1093/heapol/czz075

**Published:** 2019-07-31

**Authors:** Andrea Melberg, Alemnesh Hailemariam Mirkuzie, Tesfamichael Awoke Sisay, Mitike Molla Sisay, Karen Marie Moland

**Affiliations:** 1 Centre for International Health, University of Bergen, Årstadveien 21, N-5007 Bergen, Norway; 2 National Data Management Center for Health, Ethiopian Public Health Institute, Gulelle Arbegnoch Street, Gulele Sub City, Addis Ababa, Ethiopia; 3 School of Public Health, College of Health Sciences, Addis Ababa University, Algeria Street, Arada Sub City, Addis Ababa, Ethiopia; 4 Centre for Intervention Science in Maternal and Child Health, University of Bergen, Årstadveien 21, N-5007 Bergen, Norway

**Keywords:** Ethiopia, maternal health, indicators, MDSR, multi-sited ethnography

## Abstract

The Maternal Death Surveillance and Response system (MDSR) was implemented in Ethiopia in 2013 to record and review maternal deaths. The overall aim of the system is to identify and address gaps in order to prevent future death but, to date, around 10% of the expected number of deaths are reported. This article examines practices and reasoning involved in maternal death reporting and review practices in Ethiopia, building on the concept of ‘practical norms’. The study is based on multi-sited fieldwork at different levels of the Ethiopian health system including interviews, document analysis and observations, and has documented the politicized nature of MDSR implementation. Death reporting and review are challenged by the fact that maternal mortality is a main indicator of health system performance. Health workers and bureaucrats strive to balance conflicting demands when implementing the MDSR system: to report all deaths; to deliver perceived success in maternal mortality reduction by reporting as few deaths as possible; and to avoid personalized accountability for deaths. Fear of personal and political accountability for maternal deaths strongly influences not only reporting practices but also the care given in the study sites. Health workers report maternal deaths in ways that minimize their number and deflect responsibility for adverse outcomes. They attribute deaths to community and infrastructural factors, which are often beyond their control. The practical norms of how health workers report deaths perpetuate a skewed way of seeing problems and solutions in maternal health. On the basis of our findings, we argue that closer attention to the broader political context is needed to understand the implementation of MDSR and other surveillance systems.

**Key Messages**

- Maternal death reporting is highly politicized at all levels of the Ethiopian health system.
- Death reporting and review practices are challenged by the fact that maternal mortality is used as a major indicator of health system performance.
- Fear of personal and political accountability for maternal death strongly influences reporting and referral practices.
- Current reporting and review practices generate inaccurate knowledge for policymakers.

## Introduction

*No mother should die while giving birth*

This slogan was launched by the Ethiopian Ministry of Health in 2010 (UNICEF, 2010). It is still heard on the radio, written on ambulances, hospital registries and reports and referred to by urban policy actors and rural farmers alike a decade later. Following pressures and initiatives to eliminate maternal mortality, this article investigates how the Maternal Death Surveillance and Response (MDSR) system—brought in to identify, report and review all maternal deaths to prevent similar deaths in the future—is implemented. Based on qualitative fieldwork from one urban and one semi-rural site, this study describes how actors at different levels of the health system respond when avoidable deaths happen.

Ethiopia, located on the Horn of Africa, has a population of approximately 100 million [Central Statistical Agency (Ethiopia), 2016]. The country has shown its strong commitment to reducing maternal mortality, by from 2008 to 2016 almost tripling the number of hospitals from 112 to 316, while health centres, specialty clinics and higher clinics increased in number from 639 to 3488 (Ethiopian Public Health Institute, 2017a). Together with the training of thousands of birth care providers, the provision of skilled birth attendance has dramatically increased from 5.6% to 27.2% of births from 2000 to 2016 [Central Statistical Agency (Ethiopia), 2016]. By reducing the maternal mortality ratio (MMR) by 72% from 1990 to 2015, the country was on the brink of achieving the reductions in maternal mortality set by the Millennium Development Goals (Assefa *et al.*, 2017). Still, an estimated 13 000 women in Ethiopia die from pregnancy-related causes every year (Ethiopian Public Health Institute, 2017b). As is the case with maternal deaths worldwide, nearly all of these women could have been saved through the provision of timely low-cost, evidence-based healthcare interventions (Ronsmans and Graham, 2006).

Globally MMR is a highly politicized indictor as it is often used as the key indicator to compare the quality of clinical care, health systems in general, gender equality and women’s status between and within countries (Storeng and Béhague, 2017; Melberg *et al.*, 2018). From a measurement perspective, MMR is difficult to estimate, especially in countries without civil registration systems. There is a great deal of uncertainty and controversy between the MMRs presented by different countries, the WHO and other research institutions (Wendland, 2016). Despite the attention given to maternal mortality reduction in general, globally an estimated 300 000 women die yearly from pregnancy- and childbirth-related causes (Alkema *et al.*, 2016).

MDSR has been promoted by the World Health Organization (WHO) and other international non-governmental organizations as a key health system intervention to improve data on maternal deaths, increase accountability for maternal health and advance the quality of pregnancy and birth care (World Health Organization, 2016). The system is designed to identify and review all maternal deaths and, crucially, to respond to these deaths by interpreting the results and using them to develop appropriate policy and practical measures. These remedial actions are to be targeted at all levels of the healthcare system—to prevent future deaths from the same avoidable causes. The reviews involve determining the cause of death, and contributing factors, building on the three-delay model (delay in seeking care/delay in reaching a health facility/delay in receiving appropriate care at a health facility) (Thaddeus and Maine, 1994).

Ethiopia was among the first sub-Saharan countries to implement MDSR in 2013 (Abebe *et al.*, 2017). A comprehensive MDSR system was launched in the four largest regions of the country in 2013, and rolled out to the remaining regions in 2015. Maternal deaths were also in 2013 included as 1 out of 21 public health emergencies, which are immediately notifiable events, alongside epidemic infectious diseases such as measles and yellow fever. From 2016, perinatal deaths were also included in the system, which is currently referred to as the Maternal and Perinatal Death Surveillance and Response (MPDSR) system. MPDSR committees have been established, and review processes are conducted at all administrative levels of the Ethiopian health system, from health posts staffed by health extension workers (HEWs), through health centres, hospitals, district, zonal, regional health bureaus to the Federal Ministry of Health. According to the guidelines, facility-based deaths should be reported by birth care providers and reviews should be conducted at the health centre and hospital levels. Community-based maternal deaths should be reported by HEWs, and reviews conducted by assigned health workers at the nearby health centre. The reports produced are then reviewed by district and zonal MDSR committees which should propose action at corresponding levels and report to the regional level. The regional level should hold committee meetings to identify remedial action and develop annual reports. At the national level, all data should be collected into a database and published in annual reports reviewed by various government and non-government actors. There is a strong political commitment and a massive roll out of MPDSR committees in the country’s health facilities, to date counting about 60% of all health facilities. However, only about 10% of the maternal deaths, which would be expected according to the latest Ethiopian Demographic and Health Survey, are being reported (Ethiopian Public Health Institute, 2017b).

Policy implementation does not happen in a political vacuum. As set out in the classic policy triangle, the process of policymaking, the policy content and the policy context affect policy and its implementation (Walt *et al.*, 2008). This is indeed true for Ethiopia. Although the ruling Ethiopian People’s Revolutionary Democratic Front coalition, formally introduced democracy in 1991, scholars label the regime as authoritarian (Aalen and Tronvoll, 2009). The state has, while controlling almost all facets of society, dramatically improved health service provision and key health indicators such as infant mortality, under-five mortality and total fertility rates. The Ethiopian combination of impressive socio-economic development and elaborate administrative structures of control has been characterized as ‘developmental authoritarian’ (Matfess, 2015). This has implications both for adherence to policy implementation, and for the knowledge produced about implementation and its challenges. As Østebø *et al.* (2018) point out, social desirability bias may be particularly important when researching policy in a ‘context where there is limited freedom of speech and where non-compliance and opposition to ideas and policies promoted by the government may have serious consequences’. It is important to note that this study has been conducted alongside major political changes in Ethiopian society. Since the appointment of Prime Minister Dr Abyi Ahmed in April 2018, several reforms have been launched including the opening of the country’s political landscape, and thousands of political prisoners have been released (BBC, 2018).

Reporting and reviewing deaths is not a neutral exercise for health workers. By deciding what should be reported, when and how, we consider that health workers in implementing the MDSR system inform and actually shape policy (Lipsky, 1980). They exercise discretion over which deaths are reported, and which explanatory models are emphasized during review processes. Health workers also influence how deaths are represented in communication with bereaved families, towards the public and within the health systems in which they take place. As Lipsky (1980, p. xii) notes, ‘the routines they establish, and the devices they invent to cope with uncertainties and work pressure, effectively become the public policies they carry out’. The information reported in a death review thus represents a partial and contestable version of events. Practices in the aftermath of maternal deaths within particular health institutions become routinized in what Olivier de Sardan (2015) refers to as sets of ‘practical norms’, which very often differ substantially from policy guidelines.

This article aims to understand and draw lessons from the local implementation of maternal death surveillance, review and reporting in two selected sites in Ethiopia. More concretely, we examine how health workers do or do not report maternal deaths and the rationality behind their reporting practices. We show how the Ethiopian political context, combined with a strong political commitment to reducing maternal deaths, becomes a barrier to effective on-the-ground implementation of an ambitious and comprehensive surveillance, review and response system.

## Methods

This study is based on an ethnographic, multi-sited fieldwork (Marcus, 1995) carried out in Addis Ababa and in the surroundings of a medium-sized town with approximately 40 000 inhabitants in July/August 2018, October/November 2018 and February 2019. As we wanted to identify the local MDSR practices through which data on maternal deaths was generated, and the data flow between the local and national health system levels, we primarily used interviews and document analysis of maternal death reporting and reviews at community, health facility, woreda (district) and zonal (sub-regional) level.

A total of 35 in-depth interviews (IDIs) were conducted: 5 with men who had lost their partners to a maternal death, 4 with HEWs, 7 with health workers working in general and referral hospitals (health officers, medical doctors, nurses and midwives), 13 with health workers working in health centres (nurse and midwives) and 6 with health bureaucrats responsible for MPDSR implementation at woreda, zonal and federal levels. Study participants were purposely recruited based on their experience with maternal deaths and the MDSR system more specifically. Based on the principle of saturation, data collection was ended when no new information on the MDSR system seemed to emerge.

Interview and topic guides were prepared and were continuously updated as new issues emerged. To establish trust, initial questions were focused on maternal healthcare more generally. Then followed questions about reporting practices and norms, and exploration of the participant’s views and experiences of maternal deaths. The interviews were led by the first author, a Norwegian medical doctor experienced in qualitative research, and were conducted in Amharic or English. In the Amharic interviews, an Ethiopian co-researcher with a Master’s in Public Health acted as a translator. The interviews lasted from 30 to 105 min, and were conducted in the participants’ homes (next of kin to maternal deaths), or in a private location in their workplace (health workers). Interviews were tape-recorded and transcribed verbatim either in English or in Amharic and subsequently translated to English by research assistants experienced in transcription and translation.

Copies of written records from MPDSR reporting and review meetings were collected when available from health facilities, woreda and zonal offices where the study participants worked, to triangulate the information provided through IDI. The first author recorded the observations from the homes and health facilities visited, and recorded her general impression from the written records and reflections on the study findings in a field diary on a daily basis. These observations provided important contextual information surrounding maternal deaths and reporting practices.

After initial analysis during fieldwork, the data were analysed using thematic content analysis. Analysis was conducted on translated English transcripts by the first author, with reference to the Amharic transcripts for clarification when necessary. Following data familiarization, initial codes were identified in the interviews. These codes were first grouped into categories and then into themes. During the writing process, the themes were repeatedly assessed and refined by going back to the original dataset. Finally, the themes were narrated and representative quotes were identified.

### Ethical considerations

The institutional review board of the authors’ institutes ethically approved the study.

Before commencing interviews, the potential participant was informed about the objective of the study, that participation was voluntary, and that anonymity would be ensured. Consent, either written or by fingerprint, was obtained from all participants.

We acknowledge that maternal and perinatal deaths and their reporting constitute sensitive issues for bereaved families, front-line health workers and health bureaucrats. To preserve the study participants’ anonymity, we have chosen to refrain from giving more details on the study participants and the study locations.

## Results

### Not like other deaths

When introducing the topic of maternal death reporting during fieldwork, we were often initially met with statements about how special, sensitive and even political maternal deaths and their reporting were. Repeatedly, participants in facilities and in the communities would continue by explaining how maternal deaths had been dramatically reduced and hardly happened anymore. The special status of maternal deaths was continuously referred to; they were not like other deaths. Study participants referred to the devastating impact a maternal death would have on the household. A maternal death would leave the remaining children and partner in a difficult position. Maternal deaths were also given particular attention in health facilities. As several clinical providers pointed out, many non-maternal deaths could happen in the medical and surgical wards without much attention given to them. However, if a maternal death occurred, it would receive a lot of attention from the local community, political leaders, the hospital management and health bureaucrats.

*I think it is because the government has given place for maternal and children’s health. Death is sensitive and especially when a mother dies from pregnancy and delivery, it is difficult… When we talk about mothers, it involves all the politics, also political concerns and the government has given more attention especially the last 10 years. In addition, mostly health facility quality is measured by maternal and child health* (Clinical provider, IDI number 23).

Study participants referred to maternal deaths as political deaths. Several explanations were given for the term political, emphasizing how such deaths were given a lot of attention, and that reducing maternal deaths was a topmost political priority in Ethiopia. Urban policy actors, clinicians and rural farmers alike spontaneously referred to the slogan ‘No mother should die while giving birth’ when explaining the political nature of maternal deaths, and the gravity given to maternal deaths by political leaders. For many health workers and bureaucrats, the political importance of maternal deaths was perceived to come with pressure from community political leaders and superiors in the health hierarchy not to report any such deaths.

*Maternal death is one indicator of the quality of the health care at the woreda level, and at the community level. They (political leaders) wish no maternal deaths because they are working to limit and (make) 0 even maternal deaths. But, unpreventable maternal deaths also occur, in reality. The political leaders did not know because they are working for so many health infrastructures and health posts and health centres and ambulances that were used to limit, or (make) 0 maternal deaths. So, they did not understand that (unpreventable maternal deaths occur), because maternal deaths should simply be 0* (Health system manager, IDI number 36).

### Underreporting of maternal deaths

Although, some participants claimed that all maternal deaths in their area of responsibility (health post, health centre, hospital and woreda) were truthfully reported, there was a widespread understanding among health bureaucrats and medical doctors that maternal deaths were underreported and sometimes hidden. Several explanations for underreporting were presented, such as mere forgetfulness, work overload and a limited knowledge of the reporting procedures and guidelines outside the gynaecology and obstetrics wards. However, a pattern of selective reporting emerged, with a reluctance to report the deaths of women occurring in private health facilities, women with official residence outside the geographical catchment area of the health post, health centre, hospital, woreda or zone, and deaths occurring on referral in transportation between two health facilities.

*Most of the time the officials will say that the death happened on the way, in the ambulance, not in our health centre, not in our hospital, not in our woreda. We referred her, and then they might say that she has died in the ambulance, on the way, there is such a reason* (Health system manager, IDI number 35).

Fear of blame became central throughout data collection. Participants were generally reluctant to admit that they themselves feared being blamed when reporting maternal deaths, but would point to other health professionals, often further down in the hierarchy. Some participants mentioned difficulties in reporting maternal deaths, as the health workers in charge of reporting maternal deaths were at the same time the ones who should have prevented the very same deaths. HEWs were not only in charge of reporting maternal deaths which happened outside health facilities, but also responsible for getting women to attend health facilities to give birth, so any death outside a health facility could be seen as their own failure. Midwives and medical doctors risked external investigation and exposure of clinical malpractice in their own facility when they reported deaths. Woredas and zones could expose the infrastructural constraints in health centres and hospitals that they were supposed to improve when reporting maternal deaths. One woreda health bureaucrat explained:

*So, especially maternal deaths are not this much reported to the woreda or the region or the zones. Due to political reasons. Workers or professionals that reported these maternal deaths, they might see that they are going to be blamed by the higher positions…The health extension workers might expect that they are going to be blamed by the health centre or the health office (woreda level). We, the health office (woreda level) coordinator or officers assume that we are going to get blamed by the higher region, that’s why reports on maternal deaths are not here in our hands. As we are expected. Of course there are deaths, maternal deaths, but always there is 0 reports for our IDSR (Integrated disease surveillance and reporting) format, maternal deaths always become 0 even if we expect deaths* (Health system manager, IDI number 29).

Fear of reporting maternal deaths was also, in some, cases, the result of more or less implicit pressure from higher officials in the health system or political hierarchy. Study participants described how politics were intertwined with the everyday practices in health facilities, and how there were often conflicts over reporting practices between the political leadership and clinicians of health facilities. A maternal death would taint efforts made by politicians and higher officials to reduce maternal mortality, and harm the reputation of the health facility being questioned. The performance of health facility managers was routinely evaluated, and maternal mortality was seen as a key indicator, essential for their further career development:

*Health managers are evaluated based on their performance. If they report maternal deaths, they are not going to have an educational career. If somebody wants a master or to get promoted, he cannot report a maternal death* (Health system manager, IDI number 45).

A few clinicians reported cases of maternal deaths that were directly hindered by their superiors, or that files and reports of maternal deaths went missing within a health facility. A health bureaucrat described that he chose not to report maternal deaths that he knew of, as this would entail unofficial punishments such as not being invited for training (with the according allowance). One hospital medical director described how the reporting of health facility performance was highly politicized:

*So, everything you do, it is politics. Even it is not what you do, which is documented, it is what is acceptable to the higher bodies. And also they tell you to do that, not what’s actually happening. If there are 4 maternal deaths, they tell you to make 0. Make it 0, this was actual* (Clinical provider, IDI number 42).

### Avoiding accountability for maternal deaths

Health workers reported maternal deaths in ways that deflected responsibility for adverse outcomes, attributing death to care provided in lower-level facilities or to community and infrastructural factors beyond their control. Avoiding accountability for maternal deaths was probably linked to the political implications, but the issue of litigation also emerged as a major concern among clinicians. During the review process, personalized accountability could be avoided in the assignment of the cause of deaths. Generic causes of deaths such as infection or multi-organ failure were preferred rather than obstetric-specific causes such as puerperal sepsis or post-partum haemorrhage. In the MPDSR reporting format, maternal deaths are classified as preventable or not. While some considered nearly all maternal deaths to be preventable, others defined unpreventable deaths as those arriving too late in the health facility or those caused by infrastructural factors excluding medical malpractice. Thirdly, when deciding on the contributing delays leading to the death, on the basis of the three-delay model, several participants saw the review of maternal deaths as a way to avoid criticism of the health system and to blame maternal deaths on the woman attending care too late, by emphasizing the first and second delays.

*A mother may die, but this actual reason is not written on the discussion (MDSR) folder, they write another thing to escape; the health extension worker writes her own reason and the PHEM (public health emergency management) focal person may write his own reason in order to hide his gap. There are such experiences I found at different times in which professionals try to save themselves by putting far-flung reasons rather than writing the real findings* (Health system manager, IDI number 23).

The fear of being accused of fault for maternal deaths also resulted in what clinical providers labelled as defensive referral and medical practices. In cases where the woman’s status was seen as critical, fear of accountability among health workers would weigh heavily on clinical decision-making. In informal discussions with several senior gynaecologists, there seemed to be a consensus that dying mothers were ‘dumped’ on tertiary hospitals by regional and private hospitals. One gynaecologist working in a referral hospital explained how women with extra uterine pregnancies were referred directly to the next level of care without any required surgical procedure to stop the bleeding. This was done to avoid the risk to account for a maternal death within their facility. Another doctor explained how, in the aftermath of a case where a 9-month pregnant woman with rheumatic heart disease died from acute heart failure, he was reprimanded by the hospital management for not referring the patient to another facility. He opposed their views, as the woman needed oxygen, and would die within 10 km in an ambulance. However, he explained how decisions on care and referrals were not only about clinical judgements:

*They say I should have sent her for the responsibility not to be mine. It is not the medically right decision, it is about responsibility. If it was only about medical right and wrong, it would be fine, but it is not* (Clinical provider, IDI number 33).

For the next of kin to deceased mothers, the issue of deflecting accountability for maternal deaths among health workers was seen as an extra burden in their grieving process. Several blamed health workers present during the death for malpractice, and had considered going through the judiciary system to achieve justice. Next of kin reproached health workers and hospitals for trying to cover-up the events leading to the woman’s death rather than admitting and learning from what had actually happened. One example pertains to a maternal death occurring to a woman after an unsafe clandestine abortion. According to her husband, she was neglected for half a day in the hospital ward. Only 1 h before her death was her critical status recognized, a manual vacuum aspiration was performed and she received several units of blood. She was referred to another hospital 2 h drive away:

*The bleeding couldn’t be stopped after the (uterine) evacuation, and they referred us after she already died at night 11:00 pm. I even have the referral in my pocket, a referral for death. It is very disturbing how a professional refers after a person already died* (Family member, IDI number 39).

## Discussion

This article has described how the focus on reduction of maternal mortality, illustrated by the slogan of ‘No mother should die while giving birth’, has made maternal deaths a symbol of failure among health professionals, in the health system and in the political leadership. It has hindered accurate reporting of maternal deaths, and replaced this with strategies to avoid personal, legal and political accountability. The article has examined practices and reasoning involved in maternal death reporting and reviewing at different levels of the Ethiopian health system. By drawing on the concept of practical norms, we have revealed how health workers negotiated conflicting interests while producing data on maternal mortality and thus modified the implementation of MDSR policy. In this setting, health worker strategies involve balancing conflicting demands/concerns between reporting all deaths according to the MDSR system, and worries about revealing failures in service provision. The study has described how pressure to record improvements in maternal health combined with the fear of being held accountable influenced not only reporting and reviewing, but also care provision and referral practices.

MDSR has been framed as an important intervention to accelerate further the progress in maternal mortality reduction, with particular emphasis on high-burden countries such as Ethiopia. Studies from low-income contexts with overburdened health systems have identified significant barriers to its effective implementation (Smith *et al.*, 2017b, 2017c). These findings have sparked a debate over the suitability of MDSR in settings with high rates of maternal mortality (Koblinsky 2017; Smith *et al.*, 2017a). In this article, we will not engage in debates on whether or not MDSR implementation is the right tool to reduce maternal mortality, but we will discuss lessons from the ongoing routine implementation of the Ethiopian MDSR system.

### The political context of MDSR

MDSR has been strongly promoted by the WHO as well as donors in countries with high burdens of maternal mortality (World Health Organization, 2016). As with many other policies promoted in the field of maternal health, MDSR is highly standardized, and constitutes a ‘travelling model’ to be adopted throughout many countries (Olivier de Sardan *et al.*, 2017). The policy texts are to be implemented similarly by front-line workers in different geographical, economic, political and cultural contexts (Smith, 2001; Blystad *et al.*, 2010; Olivier de Sardan *et al.*, 2017). The WHO provides guidance for local adaptation, and tracks implementation progress across countries through implementation surveys (World Health Organization, 2016). Factors that have been reported to contribute to successful implementation of MDSR systems include political commitment, legal frameworks, the engagement of key actors and the availability of technical support from donors and professional organizations (Smith *et al.*, 2017c). Although political commitment is clearly a condition for policy adaptation, we have seen in this study how the broader political culture influences the actual implementation of the MDSR system and distorts reporting and review practices.

A large body of research has focused on enablers or barriers to well-functioning reviews in clinical implementation studies (Filippi *et al.*, 2004; Kongnyuy and van den Broek, 2008; Combs Thorsen *et al.*, 2014). Health workers’ fear of blame may compromise their willingness to participate fully and to provide accurate information in the audit process (Kongnyuy and van den Broek, 2008; Combs Thorsen *et al.*, 2014). This is indeed the case in our study, and we argue that the low proportion of expected deaths reported and reviewed by the MDSR system in Ethiopia is strongly influenced by the fact that maternal deaths have become a major indicator of health system performance, all the way from the local health post to the Federal Ministry of Health. Adherence to the regime’s ambitions on maternal mortality reduction is central to please political leadership. The challenges inherent in death reporting and reviewing, such as fear of revealing malpractice, are exacerbated by a political context where maternal mortality reduction is a top priority (Østebø *et al.*, 2018). Health workers and bureaucrats strive to balance conflicting demands from the MDSR system to report all deaths, and their own worries of disclosing failures in service provision. In addition, the lack of an adequate legal framework makes fear of litigation and other repercussions common amongst clinicians. As a result, health workers may be inclined to omit death reporting, or redefine maternal deaths as non-maternal deaths or unpreventable deaths.

When reporting causes of deaths and contributing factors, health workers in our study engaged in efforts to deflect responsibility for adverse outcomes and to cover-up poor-quality service provision. The practical norms of maternal death reporting and reviewing constitute informal regulations around routine practices, which deviate from ‘official norms’ of the MDSR guidelines (Olivier de Sardan, 2015). According to Lipsky (1980, p. xvii), negotiations during the policy implementation process and subsequent policy modifications ‘may widen the gap between policy as written and policy as performed’. How health workers select deaths to be reported, and the ways in which they do so, perpetuates a certain way of seeing problems and solutions in maternal health, leading attention away from the realties on the ground. By ‘blaming’ mothers and factors outside the control of health facilities, they obscure root health system problems and prematurely foreclose options for improving maternal healthcare (Fordyce, 2014). Policy responses based on this knowledge base may, in turn, produce remedial actions that are unfit to address and tackle systemic problems in healthcare delivery as experienced by women, children and health workers (Olivier de Sardan *et al.*, 2017).

## Conclusion

Maternal death reporting and review processes are inherently politicized, as maternal mortality has become a key indicator of development and health system functioning. This article has examined practices and reasoning involved in maternal death reporting and reviews at different levels of the Ethiopian health system. We have revealed the importance of political context when health workers negotiate conflicting interests in producing data on maternal mortality and thus modify policy at the level of implementation. On the basis of our findings, we argue that closer attention to the political context of death reporting and reviewing is necessary when introducing and implementing MDSR and similar surveillance programmes.

*Ethical approval.* The institutional review board of the Ethiopian Public Health Institute (EPHI-IRB-103-2018) and the Regional Ethical Committee Western Norway(2018/967/REK vest) ethically approved the study.

## References

[czz075-B1] Aalen L, Tronvoll K. 2009 The end of democracy? Curtailing political and civil rights in Ethiopia. Review of African Political Economy 36: 193–207.

[czz075-B2] Abebe B, Busza J, Hadush A et al 2017 ‘We identify, discuss, act and promise to prevent similar deaths’: a qualitative study of Ethiopia's Maternal Death Surveillance and Response system. BMJ Global Health 2: e000199. 10.1136/bmjgh-2016-000199 PMC5435261 28589016

[czz075-B3] Alkema L, Chou D, Hogan D et al 2016 Global, regional, and national levels and trends in maternal mortality between 1990 and 2015, with scenario-based projections to 2030: a systematic analysis by the UN Maternal Mortality Estimation Inter-Agency Group. The Lancet 387: 462–74. 10.1016/S0140-6736(15)00838-7 PMC5515236 26584737

[czz075-B4] Assefa Y, Damme WV, Williams OD, Hill PS. 2017 Successes and challenges of the millennium development goals in Ethiopia: lessons for the sustainable development goals. BMJ Global Health 2: e000318. 10.1136/bmjgh-2017-000318 PMC5656143 29081999

[czz075-B5] BBC. 2018 Abiy Ahmed: Ethiopia’s Prime Minister. London, UK.

[czz075-B6] Blystad A, van Esterik P, de Paoli MM et al 2010 Reflections on global policy documents and the WHO’s infant feeding guidelines: lessons learnt. International Breastfeeding Journal 5: 18. 20977718 10.1186/1746-4358-5-18 PMC2987852

[czz075-B7] Central Statistical Agency (Ethiopia). 2016 Ethiopia Demographic and Health Survey 2016. Addis Ababa, Ethiopia: Central Statistical Agency.

[czz075-B8] Combs Thorsen V, Sundby J, Meguid T, Malata A. 2014 Easier said than done!: methodological challenges with conducting maternal death review research in Malawi. BMC Medical Research Methodology 14: 29. 24559148 10.1186/1471-2288-14-29 PMC3946085

[czz075-B9] Ethiopian Public Health Institute. 2017a Ethiopian Emergency Obstetric and Newborn Care (EmONC) Assessment 2016. Addis Ababa, Ethiopia: Ethiopian Public Health Institute.

[czz075-B10] Ethiopian Public Health Institute. 2017b National Maternal Death Surveillance and Response (MDSR) Annual Report, 2009 EFY. Addis Ababa, Ethiopia: Ethiopian Public Health Institute.

[czz075-B11] Filippi V, Brugha R, Browne E et al 2004 Obstetric audit in resource-poor settings: lessons from a multi-country project auditing ‘near miss’ obstetrical emergencies. Health Policy and Planning 19: 57–66. 14679286 10.1093/heapol/czh007

[czz075-B12] Fordyce L. 2014 When bad mothers lose good babies: understanding fetal and infant mortality case reviews. Medical Anthropology 33: 379–94. 24964721 10.1080/01459740.2013.844696

[czz075-B13] Koblinsky M. 2017 Maternal Death Surveillance and Response: a tall order for effectiveness in resource-poor settings. Global Health, Science and Practice 5: 333–7. 10.9745/GHSP-D-17-00308 PMC5620330 28963168

[czz075-B14] Kongnyuy E, van den Broek N. 2008 The difficulties of conducting maternal death reviews in Malawi. BMC Pregnancy and Childbirth 8: 42. 18786267 10.1186/1471-2393-8-42 PMC2546364

[czz075-B15] Lipsky M. 1980 Street-Level Bureaucracy: Dilemmas of the Individual in Public Services. New York, NY: Russel Sage Foundation.

[czz075-B16] Marcus GE. 1995 Ethnography in/of the world system: the emergence of multi-sited ethnography. Annual Review of Anthropology 24: 95–117.

[czz075-B17] Matfess H. 2015 Rwanda and Ethiopia: developmental authoritarianism and the new politics of African strong men. African Studies Review 58: 181–204.

[czz075-B18] Melberg A, Diallo AH, Storeng KT, Tylleskär T, Moland KM. 2018 Policy, paperwork and ‘postographs’: global indicators and maternity care documentation in rural Burkina Faso. Social Science & Medicine (1982) 215: 28–35. 30205276 10.1016/j.socscimed.2018.09.001

[czz075-B19] Olivier de Sardan J-P. 2015 Practical norms: informal regulations within public bureaucracies (in Africa and beyond) In: De Herdt T., Olivier de Sardan J.-P. (eds), Real Governance and Practical Norms in Sub-Saharan Africa: The Game of the Rules. Oxon, UK: Routledge.

[czz075-B20] Olivier de Sardan J-P, Diarra A, Mahaman M. 2017 Travelling models and the challenge of pragmatic contexts and practical norms: the case of maternal health. Health Research Policy and Systems 15: 60. 28722553 10.1186/s12961-017-0213-9 PMC5516842

[czz075-B21] Østebø MT, Cogburn MD, Mandani AS. 2018 The silencing of political context in health research in Ethiopia: why it should be a concern. Health Policy and Planning 33: 258–70. 29165682 10.1093/heapol/czx150

[czz075-B22] Ronsmans C, Graham WJ. 2006 Maternal mortality: who, when, where, and why. Lancet (London, England) 368: 1189–200. 10.1016/S0140-6736(06)69380-X 17011946

[czz075-B23] Smith D. 2001 Texts and the ontology of organizations and institutions. Studies in Cultures, Organizations and Societies 7: 159–98.

[czz075-B24] Smith H, Ameh C, Godia P et al 2017a Authors’ response to editorial: Maternal Death Surveillance and Response: a tall order for effectiveness in resource-poor settings. Global Health: Science and Practice 5: 697–8. 10.9745/GHSP-D-17-00407 PMC5752615 29284703

[czz075-B25] Smith H, Ameh C, Godia P et al 2017b Implementing Maternal Death Surveillance and Response in Kenya: incremental progress and lessons learned. Global Health: Science and Practice 5: 345–54. 10.9745/GHSP-D-17-00130 PMC5620333 28963171

[czz075-B26] Smith H, Ameh C, Roos N, Mathai M, van den Broek N. 2017 Implementing Maternal Death Surveillance and Response: a review of lessons from country case studies. BMC Pregnancy and Childbirth 17: 233. 28716124 10.1186/s12884-017-1405-6 PMC5513145

[czz075-B27] Storeng KT, Béhague DP. 2017 ‘Guilty until proven innocent’: the contested use of maternal mortality indicators in global health. Critical Public Health 27: 163–76. 28392630 10.1080/09581596.2016.1259459 PMC5359740

[czz075-B28] Thaddeus S, Maine D. 1994 Too far to walk: maternal mortality in context. Social Science & Medicine 38: 1091–110. 8042057 10.1016/0277-9536(94)90226-7

[czz075-B29] UNICEF. 2010 *UNICEF Annual Report for Ethiopia* https://www.unicef.org/about/annualreport/files/Ethiopia_COAR_2010.pdf, accessed 8 May 2019.

[czz075-B30] Walt G, Shiffman J, Schneider H et al 2008 ‘Doing’ health policy analysis: methodological and conceptual reflections and challenges. Health Policy and Planning 23: 308–17. 18701552 10.1093/heapol/czn024 PMC2515406

[czz075-B31] Wendland CL. 2016 Estimating death: a close reading of maternal mortality metrics in Malawi In: Adams V (ed), Metrics: What Counts in Global Health. Durham and London: Duke University Press.

[czz075-B32] World Health Organization. 2016 Time to Respond: A Report on the Global Implementation of Maternal Death Surveillance and Response (MDSR). Geneva: World Health Organization.

